# Neuropsychiatric, cognitive and sexual impairment in mastocytosis patients

**DOI:** 10.1186/s13023-021-01747-y

**Published:** 2021-03-05

**Authors:** Fatma Jendoubi, Maella Severino-Freire, Mathilde Negretto, Christophe Arbus, Carle Paul, Cristina Bulai Livideanu

**Affiliations:** 1 Mastocytosis National Expert Centre (CEREMAST), Department of Dermatology, Toulouse University Hospital, 24 Chemin de Pouvourville, 31059 Toulouse, France; 2 Department of Psychiatry, Toulouse University Hospital, Tonic Inserm 1214, Toulouse, France

**Keywords:** Mastocytosis, Mast cell mediator-related symptoms, Mast cells

## Abstract

**Background:**

Mastocytosis is a rare disease characterised by the accumulation and/or proliferation of abnormal mast cells (MCs) in one or several organs. It may present with a number of different symptoms that involve various organ systems. The current study aims to assess the prevalence of MC mediator-related symptoms in a cohort of mastocytosis patients with a specific focus on neurological, psychiatric, cognitive and sexual symptoms. We also assessed the impact of the disease on patients’ professional lives. Patients were administered a validated multidimensional questionnaire to collect information on patients’ perception of the severity of their symptoms. From the questionnaires we extracted the neurological, cognitive, psychiatric and sexual symptoms and the impact of the disease on patients’ professional lives as well as their grading. The affective status was assessed using the 17-item version of the Hamilton Depression Rating Scale.

**Results:**

We included 139 patients. Mastocytosis was classified as systemic in 113 patients and cutaneous in 26 patients. The prevalence of MC mediator-related systemic symptoms was as follows: cutaneous (71%), gastro-intestinal (48%), cardio-vascular (36%), musculoskeletal (26.6%), fatigue (24%), urinary (14.4%) and respiratory (10%). Headaches and vertigo were noted in respectively 55% and 32% of patients. Irritability, episodes of memory loss and difficulty concentrating were reported in 54%, 52% and 40% of cases, respectively. Sexual impairment was noted in 24% of patients. No associations were found between neuropsychiatric/cognitive impairment and age, gender, diagnostic delay, disease form, the presence of cutaneous lesions, the level of serum and bone marrow tryptase and the presence of KIT mutation in bone marrow and/or skin. Depression was noted in 49% of patients. One in four patients reported a negative impact of the disease on their professional lives.

**Conclusion:**

This current study provides some insights regarding symptoms related to mastocytosis and their impact on patients’ professional lives.

## Introduction

Mastocytosis defines a heterogeneous group of disorders characterised by the accumulation and/or proliferation of abnormal mast cells (MCs) in one or several organs [1]. It is considered an orphan disease with a reported incidence of approximately 0.89 new cases per 100 000 inhabitants [2].

Mastocytosis occurs in two major forms: isolated cutaneous mastocytosis (ICM) and systemic mastocytosis (SM) [3].

The diagnosis of SM is established by evidence of MC infiltration in the bone marrow, liver, spleen or gastrointestinal tract. Whereas ICM is characterised by the presence of specific skin lesions and the absence of MC infiltration of internal organs [4], SM is further subdivided into indolent forms, characterised by the absence of organ dysfunction (indolent SM, smouldering SM) and advanced, life-threatening forms (aggressive SM, SM with associated clonal haematological non-mast cell lineage disease, MC leukaemia). ICM is most commonly seen in children whereas in adults, cutaneous involvement is mostly associated with SM (85%) [3].

Mastocytosis may present with several different symptoms due to the uncontrolled proliferation of MCs, the involvement of distinct organs (central nervous system, GI tract, skeleton, bone marrow) or the release of systemic mediators. The latter are referred to as MC mediator-related symptoms. MCs produce and release several clinically relevant mediators such as tryptase, histamine, leukotrienes, proteases, or heparin. Various organ systems are involved including the skin (pruritus, urticarial eruption, angioedema and flushing), the gastrointestinal tract (nausea, cramping and diarrhoea), the respiratory system (shortness of breath, chest tightness, and cough), the cardiovascular system (palpitations, severe anaphylaxis), the urinary system (pollakiuria, nycturia), the musculoskeletal system (pain) and fatigue.

In addition to MC mediator-related systemic symptoms, patients frequently report neurological (headaches, vertigo), cognitive (memory loss, difficulty concentrating), psychiatric symptoms (depression, irritability) and also sexual impairment (erectile dysfunction and dyspareunia). Such symptoms can have a major impact on patients' personal and professional lives [5].

The current study aims to assess the prevalence of MC mediator-related symptoms in a cohort of mastocytosis patients with a specific focus on neurological, psychiatric, cognitive and sexual symptoms. We also assessed the impact of the disease on patients’ professional lives.

## Materials and methods

### Study population

We included all adult patients who were part of the ongoing pathophysiological AFIRMM study (Association Française pour les Initiatives et la Recherche sur le Mastocyte et les Mastocytoses). The study was approved by the Institutional Review Board of the Necker Enfants-Malades Hospital on November 8, 2000 (RC31/17/0095) and was carried out in accordance with the Declaration of Helsinki. All patients gave their written informed consent. Patients included in the study were diagnosed with mastocytosis between January 2006 and June 2019 at the Reference Centre of Mastocytosis in Toulouse in France.

### The AFIRMM questionnaire

Patients were administered a multidimensional questionnaire designed by the AFIRMM to collect information on patients’ perception of the severity of their symptoms and how these impacted the quality of life. This validated questionnaire was designed on the basis of patient interviews to include the most commonly reported symptoms [6]. The 2.2 version used in our study includes a total of 52 symptoms forming 15 categories (skin, allergy, anaphylactic shock, flush, gastrointestinal tract, rheumatology, constitutional symptoms, cardiology, neurology/psychiatry, respiratory, urology, infection/inflammation, libido/desire, endocrinology and social life).

Patients graded each applicable disability with a score between 0 and 4 (0: Not disabling; 1: mild; 2: moderate; 3: severe; 4: intolerable).

We extracted the following symptoms and their grading from the questionnaires:

- Neurological symptoms: headache, vertigo
- Cognitive symptoms: memory loss, difficulty concentrating
- Psychiatric symptoms: irritability
- Sexual symptoms: erectile dysfunction for male and dyspareunia for female patients
- The impact on professional life: a score ranging from 1 to 4 was considered to correspond to a negative impact.

### The HAMILTON score

The affective status was assessed using the 17-item version of the Hamilton Depression Rating Scale (HDRS-17) [7]***.***

Patients were categorised as follows [8]***:***

- No depression: 0–7
- Mild depression: 8–16
- Moderate depression: 17–23
- Severe depression: ≥ 24

In addition to data retrieved from the AFIRMM questionnaire and the Hamilton score, we also extracted the following from patients’ medical files:

### Epidemiological data

- Age
- Sex
- Age at onset of symptoms, age at diagnosis and diagnostic delay (Calculated by subtracting the age at onset of symptoms from the age at diagnosis)

### Clinical data

- Mastocytosis form: All patients were diagnosed according to the WHO classification criteria [4]
- History of depression or use of anti-depressants
- Treatments targeting MC mediator-related symptoms
- Other MC mediator-related symptoms: cutaneous, cardio-vascular, gastrointestinal, urinary, respiratory and musculoskeletal symptoms and fatigue
- The presence of cutaneous lesions

### Laboratory data

- Serum and bone marrow tryptase levels
- KIT mutation status in skin and bone marrow

### Statistical analysis

Statistical analyses were performed using the R statistics software version 3.6.1 (2019–07-05). Quantitative variables were summarised using the following descriptive statistics: median with minimum, maximum. Qualitative variables were compared using the Chi-square test. Normally distributed quantitative variables were compared using Student’s *t*-test. Normally distributed quantitative variables were compared using Wilcoxon’s test. Relative risk and confidence intervals were used to analyse associations between neurological, cognitive, psychiatric, sexual symptoms, the impact on professional life and the epidemiological, clinical and laboratory data using the “EpiR” Package (Epi version 2.40).

## Results

Of the 220 patients diagnosed with mastocytosis and registered in our database, 139 had completed the AFIRMM questionnaire (78 female and 61 male).

The mean age was 48 years [range 17, 92]. Mastocytosis was classified as SM in 113 patients (Indolent: 88, advanced: 25) and ICM in 26 patients. Among patients with SM, 117 (80%) had cutaneous involvement. The median serum tryptase level was 21.8 µg/l [range 1–338] and the median medullar tryptase level was 104 µg/l [range 1.6–41,085]. KIT mutations were detected in the skin and bone marrow of 72% and 77% of patients, respectively.

At the time of the study, 56% of patients were undergoing an anti-histamine, and/or anti-leukotriene treatment. The median age at which symptoms were first observed was 48 years [range 17–92]. Median time to diagnosis was 4.5 years [range 0–35 years]. A previous medical history of depression was noted in 27% of patients and 13% of patients were undergoing pharmacological therapy for depression at the time they completed the questionnaire.

MC mediator-related systemic symptoms were noted in 85% of patients. Their prevalence was as follows: cutaneous (71%), gastro-intestinal (48%), cardio-vascular (36%), musculoskeletal (26.6%), fatigue (24%), urinary (14.4%) and respiratory (10%).

The prevalence and the severity of neuro-psychiatric, cognitive, and sexual symptoms and the impact on professional life, evaluated from the AFIRMM questionnaires, are shown in Table 1.

**Table 1:** Prevalence and severity of neuro-psychiatric, cognitive and sexual impairment and impact on professional life retrieved from the AFIRMM questionnaire

Symptom	N (%)	Severity			
		Mild (%)	Moderate (%)	Severe (%)	Intolerable (%)
Headache	65 (55)	48	37	14	1
Vertigo	38 (32)	60	29	8	3
Irritability	64 (54)	60	33	6	1
Memory loss episodes	62 (52)	70	13	17	0
Difficulty concentrating	48 (40)	56	30	14	0
Sexual impairment	27 (24)	44	33	19	4
Impact on professional life	29 (26)	20	35	38	7

Among neurological symptoms, headaches were noted in 55% of patients and vertigo in 32% of patients. Approximately one half of patients suffered psycho-cognitive impairment with irritability, episodes of memory loss and difficulty concentrating reported in 54%, 52% and 40% of cases, respectively. Neurological, psychiatric, and cognitive impairment in our cohort was considered as mild to moderate in the majority of patients. Sexual impairment was noted in 24% of patients. Out of those, sexual impairment was considered severe or intolerable in 23% of cases. Anti-histamine treatment was associated to headaches (RR 1.5952; 95% CI 1.1719–2.1715) and fatigue (RR 2.1217; 95% CI 1.1236–4.0063) but not to other neurological, psychiatric and cognitive symptoms.

No associations were found between neuropsychiatric/cognitive impairment and age, gender, diagnostic delay, disease form (ICM or SM), the presence of cutaneous lesions, the level of serum and medullar tryptase and the presence of KIT mutation in BM and/or skin (Table 2).

**Table 2:** Association between symptoms and epidemiological, clinical and laboratory parameters

	Depression	Irritability	Neurological symptoms	Cognitive symptoms	Sexual symptoms
Median age RR (95% CI)	1.2539 (0.8511–1.8475)	0.9307 (0.6903–1.2549)	0.9179 (0.7107–1.1855)	1.1670 (0.8886–1.5327)	1.2591 (0.6741–2.3516)
Frequency of male RR (95% CI)	0.6774 (0.4508–1.0177)	1.2212 (0.9079–1.6427)	0.8206 (0.6265–1.0748)	1.0521 (0.8031–1.3783)	1.1754 (0.6308–2.1905)
Median diagnostic delay RR (95% CI)	1.0833 (0.7339–1.5989)	0.9307 (0.6903–1.2549)	1.0940 (0.8456–1.4153)	0.9805 (0.7484–1.2845)	0.9375 (0.5028–1.7480)
Frequency of cutaneous lesions RR (95% CI)	1.1761 (0.7263–1.9045)	1.0762 (0.7259–1.5955)	1.2107 (0.9047–1.6203)	1.2483 (0.9257–1.6834)	0.9060 (0.3587–2.2883)
Mastocytosis form RR (95% CI)	1.3578 (0.7589–2.4293)	0.8290 (0.5963–1.1525)	0.9983 (0.7203–1.3836)	0.9809 (0.6987–1.3770)	1.2000 (0.5126–2.8092)
Serum tryptase level RR (95% CI)	1.3102 (0.8051–2.1322)	1.0190 (0.7295–1.4233)	1.0989 (0.8198–1.4730)	1.1585 (0.8453–1.5878)	0.7814 (0.4136–1.4761)
Bone marrow tryptase level RR (95% CI)	0.9863 (0.6554–1.4843)	1.0470 (0.7427–1.4759)	1.0628 (0.7973–1.4165)	1.1596 (0.8355–1.6094)	0.7429 (0.3926–1.4058)
KIT mutation status in BM RR (95% CI)	1.1780 (0.7063–1.9650)	0.9278 (0.6591–1.3060)	0.9968 (0.7378–1.3467	1.0833 (0.7641–1.5358)	0.7704 (0.3856–1.5392)
KIT mutation frequency in skin RR (95% CI)	0.9267 (0.6020–1.4265)	0.8741 (0.6377–1.1982)	0.9135 (0.6940–1.2023)	0.9687 (0.7170–1.3086)	1.1560 (0.5473–2.4418)

There was no association between neuropsychiatric / cognitive impairment and the presence of MC mediator-related systemic symptoms, except for headaches (RR 1.9897; 95% CI 1.0112–3.9152).

The Hamilton questionnaire was administered to 108/139 patients (78%). The median score was 8.63 [range 0, 30]. Depression was noted in almost half of patients (49%) and was mild in 33.3% of patients and moderate and severe in 7.4% and 5.6% of patients, respectively. A total of seven patients with a Hamilton score < 8 (no depression) were undergoing antidepressant therapy (6.5%).

Patients with urinary symptoms had a higher prevalence of depression (60%) compared to patients without such symptoms (30%) (RR 1.5060; 95% CI 1.0109–2.2434). The same finding was observed for gastro-intestinal symptoms (48% vs 22%; RR 1.7398; 95% CI 1.1376–2.6606) and fatigue (50% Vs 30%; RR 1.5678; 95% CI 1.0891–2.2569).

Depression was also associated with cognitive (RR 4.2524; 95% CI 2.0040–9.0233), sexual (RR 1.9076; 95% CI 1.3546–2.6863) and neurological symptoms (RR 2.6699; 95% CI 1.4670–4.8592).

One in 4 patients declared that the disease had a negative impact on their professional life. This effect was considered severe or intolerable in 45% of cases.

**Fig. 1:**
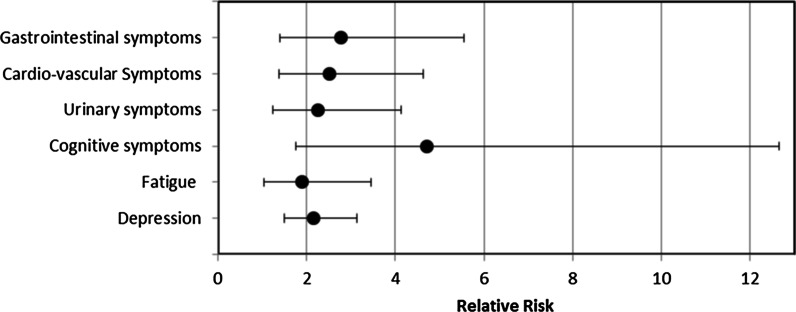
The relation between the impact on professional life and gastro-intestinal, cardiovascular, urinary tract and cognitive symptoms, depression and fatigue

This impact on professional life was more prevalent in patients with gastro-intestinal symptoms, cardiovascular symptoms, urinary tract symptoms, cognitive symptoms, depression and fatigue (Fig. 1).

## Discussion

Our current study indicates that mastocytosis patients have a high prevalence of potentially disabling symptoms. Among patients not receiving anti-depressant therapies, some 49% were depressed according to an analysis based on the Hamilton score. Headaches were also noted in one out of two patients of our cohort and were associated to anti-histamine treatment. Episodes of vertigo were noted in one out of every three patients. Cognitive and sexual impairment were also prevalent, affecting up to 50% and 25% of patients, respectively. No association between the latter symptoms and anti-histamine treatment was noted. In addition, one in four patients reported a negative impact on their professional life.

Depression was associated with cognitive, sexual, urinary, gastro-intestinal symptoms and fatigue. However, no associations were detected between depression and cutaneous, respiratory, and cardio-vascular symptoms. As the majority of patients were receiving anti-histamines, and *montelukast* treatment at the time of completing the questionnaire, this may have reduced our ability to detect an association.

The prevalence of neurological, cognitive, sexual, and psychiatric symptoms (including depression) was similar in patients with cutaneous and systemic mastocytosis. Symptoms were not influenced by indicators of total MC burden such as serum or medullar tryptase level [9–11]. There were not associations between symptoms and the presence of KIT mutations.

There is limited data about the frequency of neuro-cognitive, psychiatric, and sexual impairment in mastocytosis patients. Two previous studies reported the prevalence of neurological symptoms in mastocytosis patients [12, 13]. Headaches were the most prevalent symptom affecting some 35 to 56% of patients. Experiencing a headache was associated with an increased prevalence of other MC mediator-related systemic symptoms [12].

Cognitive impairment was first reported by Soter et al*.* in five out of eight mastocytosis patients [14]. In another small study, ten patients underwent psychiatric interviews and a battery of psychological testing. Out of these, eight presented with cognitive and affective changes categorised as mixed organic brain syndrome [15]. In another study 22 out of 57 mastocytosis patients (38.6%) presented with memory/concentration impairment. This cognitive impairment was not related to depression, age or the type of mastocytosis [16]. Our current study, of a larger cohort (n = 139), reports a higher prevalence of cognitive impairments such as memory loss and difficulty concentrating in 52% and 40% of patients, respectively. We also observed an association between cognitive symptoms and depression, urinary and gastro-intestinal symptoms, and fatigue but not with age. Although depression and cognitive impairments are common co-morbidities, understanding their relationship remains limited [17]***.***

In terms of mood disorders, Roger et al*.* diagnosed depression from psychiatric interviews in four mastocytosis patients out of ten [15]. This was further investigated in two other studies. The first of these studies calculated a HAMILTON score for 88 patients and found that 75% of patients had scores of ≥ 10 [6]. The second study, involving 288 patients, reported 56% of patients with a HAMILTON Score between 8 and 22 which was categorised as mild-moderate depression based on the cut-offs used in that study [18]. In contrast to our study, these two previous studies did not assess associations between depression and other mastocytosis symptoms. The Moura et al*.* study showed that *mastinib* treatment, a tyrosine kinase inhibitor which decreases disease burden, also improved depression scores. Interestingly, this improvement was unrelated to the improvement in overall quality of life. This is also consistent with a phase 2a multicentre study which evaluated the effect of *masitinib* treatment in 25 mastocytosis patients and showed a > 50% improvement in Hamilton scores compared to baseline [19]. Improvement in depression could be related to the inhibitory effect of *masitinib* on MC activation which would support the assumption that depression is a systemic manifestation of the disease [20].

The frequency of depression in mastocytosis patients appears to be higher to what might be expected in the general population (7%) and in patients with advanced chronic conditions such as cancer (10–15%) and diabetes (14–25%) [21–24].

This raises the question of whether neurological, affective, and cognitive impairment is the result of primitive MC infiltration of the brain or the effects of MC mediators.

Boddaert et al. reported morphological brain abnormalities consisting on abnormal punctuated white matter hyper signals in 49% of mastocytosis patients with psycho-cognitive complaints. As no histological diagnosis was done in this study, no evidence of MCs infiltration of the brain could be found. The authors suggested the implication of MC mediators without MC infiltrates in the recruitment and the proliferation of inflammatory cells resulting in neuroimaging abnormalities and neuro-cognitive symptoms [25].

Indeed some experimental evidence suggests that MC mediators may contribute to headaches, depression, and cognitive impairment.

Degranulation of dural mast cells induces activation of the trigeminal pain pathway believed to underpin migraine headaches [25]. Histamine, leukotrienes, TNF-alpha, IL-6 and endothelin-1 released from MCs have been implicated in the pathophysiology of migraines [26–28]***.***

A study of fifty-four mastocytosis patients has also suggested a role for MCs in the tryptophan catabolic pathway which is involved in depression. Alterations in tryptophan and serotonin metabolism, which are implicated in depression pathogenesis, have also been demonstrated in mastocytosis patients [29]

The implication of pro-inflammatory cytokines and MC-derived inflammatory mediators in neuro-inflammation and neurodegeneration could also explain the cognitive impairment in mastocytosis [30]. The degranulation of MCs and the release of histamine [31] activate microglia through histamine receptors H1 and H4 and release neurotoxic mediators such as IL-1b, TNF-alpha, IL-6, and nitric oxide (NO) [32]. These pro-inflammatory mediators directly induce neuronal death in the brain. Inflammatory cytokines such as IL-1b are known to induce neurodegeneration [33]***.***

We therefore suspect that these symptoms are the result of mast cell activation and the systemic release of mediators rather than due to the proliferation or infiltration of MCs. This hypothesis is also sustained by the lack of impact of the type of mastocytosis (cutaneous or systemic) on these symptoms.

Regarding the impact of the disease on patients’ professional lives (which affects 26% of patients in our study), it appears to be noticeably higher than what had been reported in one previous study (15%) [34]. This emphasises the importance of disease burden in mastocytosis patients. In our cohort, this impact was associated with psycho-cognitive impairment, gastro-intestinal, cardiovascular, and urinary symptoms, and fatigue.

The patient's perception of disability was assessed in a cohort of 62 patients with a confirmed mastocytosis diagnosis. Perceived handicap was noted in 87% of patients and was scored as moderate to severe in 52% of patients. In the same study, psychological, sexual, cognitive and neurological symptoms were found to be key contributors to disability in mastocytosis [6].

Our study is the first, to our knowledge, to simultaneously evaluate the neurological, cognitive, and emotional impacts of mastocytosis, which highlights the high degree of patient distress. There is an urgent need for physicians to recognise and treat neuro-psychiatric and cognitive symptoms associated with mastocytosis and to better understand the impact of such symptoms on the lives of their patients.

Our study has some limitations which are essentially due to its retrospective and monocentric nature. The available clinical data also did not allow us to assess the different headache subtypes. To assess depression, we used a validated measure and not a structured diagnostic interview which is generally considered the gold standard to detect depression.

We also only assessed subjective cognitive impairment and did not use objective instruments to evaluate this type of impairment in our mastocytosis patients.

## Conclusion

Our current study provides some insights regarding symptoms related to mastocytosis and their impact on patients’ professional lives. Further research is needed to evaluate the link between MCs and neuro-psychiatric, cognitive, and sexual symptoms.

## Data Availability

The datasets generated and/or analysed during the current study are not publicly available due data size but are available from the corresponding author on reasonable request.

## Abbreviations

MCs: Mast cells
MC: Mast cell
ICM: Isolated cutaneous mastocytosis
SM: Systemic mastocytosis
AFIRMM: Association Française pour les Initiatives et la Recherche sur le Mastocyte et les Mastocytoses
HDRS-17: 17-Item version of the Hamilton Depression Rating Scale
BM: Bone marrow
NO: Nitric oxide
